# MBTPS1 regulates proliferation of colorectal cancer primarily through its action on sterol regulatory element-binding proteins

**DOI:** 10.3389/fonc.2022.1004014

**Published:** 2022-10-10

**Authors:** Liat H. Hartal-Benishay, Esraa Saadi, Shir Toubiana, Lior Shaked, Maya Lalzar, Ossama Abu Hatoum, Sharon Tal, Sara Selig, Liza Barki-Harrington

**Affiliations:** ^1^ Department of Human Biology, Faculty of Natural Sciences, University of Haifa, Haifa, Israel; ^2^ Department of Genetics and Developmental Biology, Rappaport Faculty of Medicine and Research Institute, Technion, Haifa, Israel; ^3^ Bioinformatics Service Unit, Faculty of Natural Sciences, University of Haifa, Haifa, Israel; ^4^ Department of Surgery, Ha’emek Medical Center, Afula, Israel; ^5^ Department of Medicine, Rappaport Faculty of Medicine and Research Institute, Technion, Haifa, Israel; ^6^ Laboratory of Molecular Medicine, Rambam Health Care Campus, Haifa, Israel

**Keywords:** MBTPS1, SKI-1/S1P, site-1 protease, colon cancer, SREBP, CRISPR/Cas9, HT-29, lipid metabolism

## Abstract

Among the main metabolic pathways implicated in cancer cell proliferation are those of cholesterol and fatty acid synthesis, both of which are tightly regulated by sterol regulatory element-binding proteins (SREBPs). SREBPs are activated through specific cleavage by membrane-bound transcription factor protease 1 (MBTPS1), a serine protease that cleaves additional substrates (ATF6, BDNF, CREBs and somatostatin), some of which are also implicated in cell proliferation. The goal of this study was to determine whether MBTPS1 may serve as a master regulator in proliferation of colorectal cancer (CRC). Tumors from CRC patients showed variable levels of MBTPS1 mRNA, which were in positive correlation with the levels of SREBPs and ATF6, and in reverse correlation with BDNF levels. Chemical inhibition of MBTPS1 activity in two CRC-derived cell lines resulted in a marked decrease in the levels of SREBPs, but not of its other substrates and a marked decrease in cell proliferation, which suggested that MBTPS1 activity is critical for proliferation of these cells. In accordance, CRISPR/Cas9 targeted knockout (KO) of the *MBTPS1* gene resulted in the survival of only a single clone that presented a phenotype of severely attenuated proliferation and marked downregulation of several energy metabolism pathways. We further showed that survival of the MBTPS1 KO clone was dependent upon significant upregulation of the type-1 interferon pathway, the inhibition of which halted proliferation entirely. Finally, rescue of the MBTPS1 KO cells, resulted in partial restoration of MBTPS1 levels, which was in accordance with partial recovery in proliferation and in SREBP levels. These finding suggest that MBTPS1 plays a critical role in regulating colon cancer proliferation primarily through SREBP-associated lipid metabolism, and as such may serve as a possible therapeutic target in CRC.

## Introduction

Proliferation of cancer cells is dependent upon activation or enhancement of specific metabolic pathways in order to supply their growing energetic needs. Two major pathways that are often deregulated in cancers cells are those of cholesterol and fatty acid synthesis (1). Cholesterol is an essential molecule for membrane and hormone biosynthesis and multiple *in vitro* studies have demonstrated that inhibition of HMG-reductase, the rate limiting enzyme of cholesterol synthesis, is detrimental to cancer cell growth (Reviewed in (2). However, clinical studies that tested the effect of statins-HMG CoA reductase inhibitors as potential anti-cancer drugs have so far been inconclusive (3). Increased fatty acid synthesis and uptake have also been identified as promoting tumor growth and several inhibitors of enzymes in these pathways are being tested, with no clear results thus far (1).

One of the most important regulators of synthesis and uptake of cholesterol, fatty acids, triglycerides and phospholipids are a family of sterol regulatory element-binding proteins (SREBPs) transcription factors (4). Two main isoforms of SREBPs, SREBP1 and SREBP2 (encoded by the genes *SREBF1* and *SREBF2*, respectively) are synthesized as inactive precursors that are anchored to the membranes of the ER and nuclear envelope, and activated through cleavage by MBTPS1 (membrane-bound transcription factor protease, also known as site-1 protease or SKI-1). The cleavage of SREBPs facilitates their localization to the nucleus where they activate transcription of target genes such as the low density lipoprotein (LDL) receptor and 3-hydroxy-3-methylglutaryl-CoA (HMG-CoA) reductase - the rate limiting enzyme in cholesterol synthesis (4).

MBTPS1 is a calcium-dependent serine protease that is encoded by the *MBTPS1* gene, synthesized in the endoplasmic reticulum (ER) as an inactive precursor that becomes active upon autocatalytic processing in the Golgi apparatus (5–8). Two MBTPS1 substrates, MBTPS1 itself, and the membrane-bound precursor of N-acetylglucosamine (GlcNac)-1 phosphotransferase (9), are constitutively cleaved by MBTPS1, while its other substrates are cleaved upon intracellular signals. In addition to SREBPs, MBTPS1 cleaves and activates several transcription factors that are critical for various cellular functions. These include ATF6 (10), cyclic AMP-responsive element-binding proteins (CREB) 3 and 4 (11, 12), the pro-form of the secretory brain-derived neurotrophic factor (BDNF) (8, 13) and pro-somatostatin (14). ATF6 is one of the three ER-resident proteins that regulate the unfolded protein response (UPR) and is activated upon ER stress signaling [reviewed in (15)]. ATF6 is also activated directly by specific lipids (16), and interacts with activated peroxisome proliferator-activated receptor a (PPARα), a key transcription factor that controls fatty acid oxidation in the liver (17). Like SREBPs and ATF6, CREB3 is also cleaved by MBTPS1 in the Golgi apparatus, and its subsequent translocation to the nucleus has multiple tissue-dependent roles including acute cell response, lipid metabolism, survival and differentiation [reviewed in (18)]. In contrast to SREBPs, ATF6 and CREBs the physiological significance of BDNF and somatostatin cleavage by MBTPS1 remains unclear.

Previous studies indicate that several of MBTPS1 downstream targets are implicated in growth of colorectal cancer (CRC) cells. Knockdown of SREBPs in CRC-derived cells was shown to significantly hamper the rate of fatty acid synthesis (19), cell proliferation and the ability of the cells to form spheroids, as well as to inhibit xenograft tumor growth and decrease the expression of genes associated with cancer stemness (20). In another study, inhibition of the SREBP1 pathway suppressed growth and lipogenesis of colon cancer xenografts (21). ATF6 was also found to be linked to CRC by upregulating the inhibitor of protein phosphatase 2A (CIP2A), an oncogene that increases cancer cell survival (22, 23). Mice with intestinal epithelial expression of the active form of ATF6 developed spontaneous colon adenomas at 12 weeks of age, and in CRC patients increased ATF6 expression was associated with reduced time of disease-free survival (23). The levels of another MBTPS1 target, BDNF, were also found to be elevated in human CRC samples where its presence was associated with reduced apoptosis of cancer cells (24). Increased BDNF levels also enhanced migration of colon cancer cells (25).

MBTPS1 belongs to the family of proprotein convertases (PCs), some of which have been implicated in cancer cell proliferation. PC members PC2 and PC3 were found to be expressed in adrenal tumors (26), and elevated in small cell lung carcinoma (SCLC), while the PCs furin and PACE4 were described as highly expressed in non-small lung carcinoma (NSCLC) (27). Several studies also showed that the expression of some PCs correlates with rapid growth, invasiveness or metastatic potential of several tumor-derived cell lines [reviewed in (28)]. However, to date, only a few studies specifically link MBTPS1 to tumorigenesis. Weiss et al. demonstrated that inhibition of MBTPS1 by a small peptide inhibitor suppressed the growth of melanoma cells (29), and Caruana et al. found that treatment of glioblastoma cells with a chemical MBTPS1 inhibitor decreased cell viability, induced apoptosis and downregulated cholesterol and fatty acid biosynthesis pathways (30). Since MBTPS1 is upstream to numerous factors implicated in CRC prosperity, we used a combined chemical and genetic approach to examine its specific role in regulating CRC proliferation.

## Materials and methods

### Materials

The MBTPS1 inhibitor PF-429242 dihydrochloride (Cat. # SML0667) and Poly(I:C) (Cat #. P1038) were purchased from Sigma Aldrich (Merck, Israel). STAT1 inhibitor Fludarabine (Cat. # 14128), gift of Prof. Amiram Ariel, was from Cayman Chemical (Ann Arbor, MI, USA). Apoptosis was measured using the MEBCYTO-Apoptosis kit (Annexin V-FITC Kit) from Medical & Biological Laboratories (Nagano, Japan). All cell culture media, fetal bovine serum and antibiotics were from Biological Industries (Beit HaEmek, Israel). All other materials were standard laboratory grade.

### Patients and RNA extraction

Biopsies were obtained from patients diagnosed with adenocarcinoma of the colon or rectum at Ha’Emek Medical Center, Afula, Israel. Surgery was performed on all patients prior to any neoadjuvant treatment by radiation, and samples were obtained according to the Declaration of Helsinki as revised in 2008 (Ha’Emek Medical Center- 0049–19). Samples were submerged in approximately 5-10 volumes of RNA SAVE solution (Cat. # 01-891-1A, Sartorius, Israel) and kept at room temperature for 24 hours, after which they were frozen at -80°C pending analysis. For RNA extraction, the tissue was placed on top of a closed ice-filled glass Petri dish, washed with ice-cold PBS and cut into small pieces. RNA was then extracted using Quick-RNA™ Miniprep Plus Kit (Cat. # R1058, Zymo Research), according to the manufacturer’s instructions. The concentration of RNA was determined using Nanodrop-1000 (Thermo Scientific), and cDNA was synthesized using High-Capacity cDNA Reverse Transcription Kit (Cat. #4374966, Applied Biosystems).

### Quantitative RT- PCR

RT-qPCR was carried out on an Applied Biosystems StepOnePlus Real-Time PCR system with Fast SYBR Green Master Mix (Cat # 4385612, Applied Biosystems). Analysis was carried out by the ΔΔCt method using the β2 microglobulin as the reference gene. Results were analyzed using StepOne software (Applied Biosystems). Average relative quantification (RQ) values were calculated for each tumor and compared to the levels obtained from the same patient’s normal tissue.

### List of RT-qPCR primers

**Table:** 

Gene	Forward	Reverse
ATF6	5’-GGAGCCACTGAAGGAAGATAA G-3’	5’-GTGCTGCTGGAAGCAATAAAG-3’
BDNF	5’-GGTGCTGTTGTCAT GCT TTA C-3’	5’-CTCTACTCCCTGTGGGAACTAA-3’
CREB3	5’-GTAGAGGGACAGTGGATAGGT-3’	5’-TTGGGACAACTACGGAAAGG-3’
HMGCS1	5’-AAGAAAACACTCCAATTCTCTTCC CT-3’	5’-GTACACATCTTCAGTATATGGTTCCC-3’
HMGCS2	5’-CACCAACAAGGACCTGGATAA-3’	5’-CCATTGTGAGTGGAGAGGTAAA-3’
MBTPS1	5’-GGGAGTGCCAAGGATTTG C-3’	5’-GCGTCCAAAAACCAAGATGTG-3’
PPRG	5’-GCCTGCATCTCCACCTTATTA-3’	5’-ATCTCCACAGACACGACATTC-3’
PPRGC1A	5’-TGAACTGAGGGACAGTGATITTC-3’	5’-CCCAAGGGTAGCTCAGTTTAT C-3
SREBF1	5’-GAGCCATGGATTGCACTTTC-3’	5’-AGCATAGGGTGGGTCAAATAG-3’
SREBF2	5’-CTGTAGCGTCTTGATTCTCTCC-3’	5’-CCTGGCTGTCCTGTGTAATAA-3’
SST	5’-TGGAAGACTTTCACATCCTGTT-3’	5’-CGCTGAAGACTTGGAGGATTAG-3’

### Cell culture

Human epithelial adenocarcinoma HT-29 and HCT-116 cells, obtained from the American Type Culture Collection repository (HTB-38, Manass, VA, USA). HT-29 cells were cultured in RPMI medium and HCT-116 in DMEM and both were supplemented with 10% heat-inactivated fetal bovine serum and 100 U/mL penicillin and streptomycin (Biological Industries, Beit HaEmek, Israel).

### Microscopy

30,000 cells were seeded on 13-mm^2^ glass coverslips, grown for 72 h prior to imaging, and mounted onto glass slides using Mowiol (Cat. # 81381 Sigma Aldrich, Saint Louis, MI, USA) for visualization by the Nikon Eclipse Ti2-E inverted wide-field fluorescent/brightfield microscope with a Differential Interference Contrast (DIC) module. All images were acquired using the same exposure conditions at the Bioimaging Unit, University of Haifa.

### Proliferation and apoptosis assays

For live cell tracking experiments, 30,000 HT-29 cells were seeded into 24-well dishes that were placed in the IncuCyte^®^ ZOOM live-cell analysis system (Essen Bioscience, Ann Arbor, MI, USA) or the Cytation 5 Cell imaging Multi-Mode Reader (Agilent Bio Tek Imaging, Santa Clara CA, USA) for various durations, and snapshots were taken every 60 min. Percent confluency was analyzed over time using the IncuCyte^®^ ZOOM Software or the Gene 5 software, respectively at the Biomedical Core Facility, Rappaport Faculty of Medicine, or the RBNI both in the Technion Israel Institute of Technology, Israel. Each experimental condition contained three repeats and was carried out in a minimum of two biological repeats (number of biological repeats for each experiment is indicated in the respective figure legends). Cell proliferation was also assessed under the same conditions using the 3-bis-(2-methoxy-4-nitro-5-sulfophenyl)-(2*H*)-tetrazolium-5-carboxanilide (XTT) kit (Biological Industries, Beit HaEmek, Israel). Each experimental point contained 4–8 technical repeats and was performed in a minimum of three biological repeats.

Apoptosis measurements were done using IncuCyte^®^ ZOOM live tracking system. 10,000 or 5,000 cells were seeded in 96-well dishes and cells were treated with or without PF-429242 immediately before tracking commenced. Apoptosis was measured using the IncuCyte^®^ Caspase3/7 Green Apoptosis Reagent (Cat. # 4440, Essen Bioscience).

### Western blotting

Total cell lysates were processed for western blotting as described (31). Nitrocellulose membranes were incubated with primary antibodies at a dilution of 1:500–1000. The following antibodies were used: Rabbit polyclonal anti phospho-STAT-1 (Cat. #9167, clone 58D6) from Cell Signaling Technology, and Mouse monoclonal anti-Actin (clone 4) from MP Biomedicals, and Mouse monoclonal anti-SREBP1 (2A4, Cat# SC-13551) from Santa Cruz Biotechnology Inc. Proteins were visualized by a WesternBright ECL (AdvanstaMenlo Park, CA, USA), quantified using Amersham Imager 600 (GE, Buckinghamshire, UK) and analyzed using Quantity One -1D analysis software.

### Gene editing

CRISPR/cas9 mediated knockout of the *MBTPS1* gene in HT-29 cells was carried out as following: a guide RNA (gRNA) targeting a coding region in exon 5 of *MBTPS1* (5’-ATCGTCCAGCGTTCGCTCGT-3’) was designed using the Optimized CRISPR Design online tool (http://crispr.mit.edu) and cloned into pSpCas9(BB)−2A-GFP (PX458), a gift from Feng Zhang (Addgene plasmid #48138) (32). This plasmid was introduced into HT-29 cells by electroporation. GFP-positive single cells were sorted the following day into 96-well plates using the FACS Aria IIIu cell sorter, and expanded to obtain individual clones. Genomic DNA, extracted from multiple clones, was subjected to PCR using *MBTPS1*-specific primers (Fwd 5’- TTTTCTGTGGGTCCCAGG -3’ and Rev 5’- TCCTGAAGTGCTACCTCC -3’), designed to amplify a 385bp region including the gRNA target site. PCR products were Sanger-sequenced to detect clones in which the open reading frame (ORF) of all *MBTPS1* alleles was disrupted by non-homologous end joining (NHEJ).

### Fluorescent *in situ hybridization (FISH)*

HT-29 cells were treated with colcemid, harvested by trypsinization, treated with hypotonic solution and fixed with methanol/acetic acid (3:1). Cells were then dropped on slides and hybridized by a standard FISH protocol to a probe generated from BAC clone RP11-274I19 (BACPAC Genomics, Emeryville, California) which overlaps with the *MBTPS1* gene. Probe DNA was labeled with dUTP-digoxigenin and detected with anti-Dig-Rhodamine. DNA was stained with DAPI. Nuclei and chromosomes were visualized on a BX50 microscope (Olympus). Images were captured with an Olympus DP70 camera controlled by DP controller software (Olympus).

### Viral infection

A lentiviral plasmid containing the ORF of the *MBTPS1* gene (Cat# 2819001, abm) was introduced by transduction into the HT-29 MBTPS1 KO clone according to manufacturer’s instructions. In short, the viral packaging cell line HEK-293T was used to generate lentiviral particles by co-transfection of the expression vector together with VSVG and pMD2 plasmids. Transfection was performed using Lipofectamine™ 3000 Reagent (L3000-008, Invitrogen). Forty-eight hours post transfection the supernatant containing the viruses was collected and filtered through a 0.45-μm PVDF filter. Viruses were used to infect the HT-29 cells in the presence of 6µg/ml polybrene (Millipore, TR-1003-G). Selection for cells that incorporated the viral sequence was performed with puromycin.

### CXCL1 ELISA

100,000 HT-29 or MBTPS1 KO cells were seeded in 6-well dishes. One day after seeding, cells were washed twice with warm PBS and fresh media containing 50 μg/ml Poly(I:C) was added for 24h. The amount of CXCL1 was determined using the Human CXCL1/GROα DuoSet Elisa (# DY275-05, R&D Systems) according to the manufacturer’s instructions.

### Transcriptome analyses and statistics

Total RNA was prepared in three biological repeats using the Quick-RNA MiniPrep kit (Cat. # ZR-R10554, Zymo Research). Library preparation was performed using NEBNext Ultra RNA library Prep kit for Illumina (Cat. # E7530L, ThermoFischer Scientific, Waltham, MA USA), according to the manufacturer’s protocol. Sequencing (single-read, 50bp) was carried out using the Illumina HiSeq 2500 at the TGC-Technion Genome center (Technion, Haifa, Israel). Sequence reads were aligned to the human reference genome version GRCh37 using Tophat (2.0.9). Gene expression levels were quantified using Htseq-count (0.6.1-py2.7) and differential expression was analyzed using EdgeR (3.2.4). Differential expression was considered significant for *P*-value< 0.05. The differentially expressed (DE) gene set was subjected to gene-set enrichment analysis using ENRICHR [accessed Jan 2022 (33)] considering gene ontology biological processes database. Cutoff for significant enrichment was adjusted based on *P* value<0.05 (**Table S1**). For each significantly enriched pathway, the percentage of DE genes in the pathway was calculated. In addition, the trend toward up or down regulation was expressed as a z-score, calculated as up−downtotal DE  .

A network describing the overlap in genes between significantly enriched pathways was calculated using R package ‘igraph’ (version 1.2.7) based on pairwise Jaccard distances matrix between pathways. Pathways selected for network analysis included those for which the z-score value was >|2|. The resulting network was exported and visualized in Cytoscape (version 3.9.0).

For the correlation studies, Pearson’s correlation coefficient (r) was used to measure the strength of correlations between the different variables, *P*<0.05 was considered significant.

## Results

### Correlation between mRNA expression of MBTPS1 and its downstream targets in tumors of CRC patients

**Figure 1:**
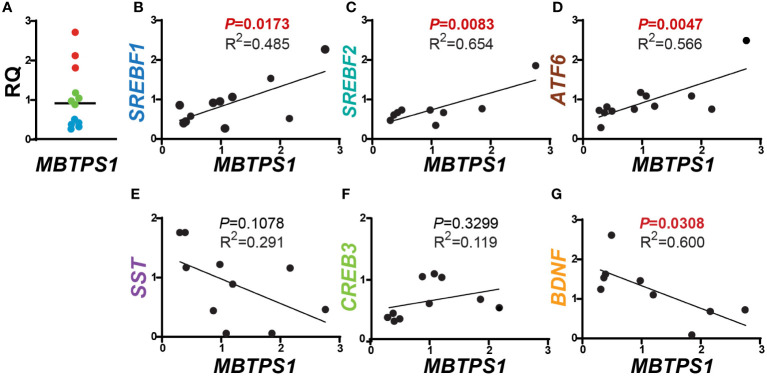
Correlations between *MBTPS1* levels and its downstream targets in human CRC. **(A)** Relative *MBTPS1* mRNA levels in tumors compared to normal surrounding tissue obtained from the same patient (*n*=12 patients). Note that while in some patients the levels of *MBTPS1* are unchanged (green circles), in others the levels are markedly elevated (red circles) or markedly reduced (light blue circles). **(B–D)** Positive correlation between *MBTPS1* and three MBTPS1 targets: *SREBF1* (r = 0.6963, *P* = 0.0173), *SREBF2* (r = 0.8086, *P* =0.0083) and *ATF6* (r = 0.7526, *P* = 0.0047). **(E, F)** No correlation between *MBTPS1* and *SST* (r = -0.5691, *P* = 0.1078) or *CREB3* (r = 0.3443, *P* = 0.3299). **(G)** A negative correlation between *MBTPS1* and *BDNF* (r = -0.7138, *P* = 0.0308).

In order to test the hypothesis that MBTPS1 is directly involved in CRC proliferation, we first determined its expression in colorectal tumors in comparison to normal surrounding tissue from patients diagnosed with low or moderate colorectal adenocarcinoma. Due to the lack of satisfactory commercial antibodies against MBTPS1, we were unable to adequately assess its protein levels and therefore measured mRNA levels. Three categories of *MBTPS1* expression were noticeable among the tumor samples (**Figure 1A**). In some patients, *MBTPS1* expression was comparable between tumor samples and normal surrounding tissue (**Figure 1A**, green dots). In the remaining tumor samples, we found that *MBTPS1* levels were either significantly decreased (**Figure 1A**, light blue dots) or significantly increased (**Figure 1A**, red dots).

We next sought to determine whether the variability in *MBTPS1* expression affects the mRNA levels of its known downstream targets. As depicted in **Figure 1**, we found a positive correlation between the levels of *MBTPS1* and *SREBF1*, *SREBF2* (the SREBP genes), and *ATF6* (**Figures 1B–D**), i.e. samples with low levels of *MBTPS1* mRNA showed low expression levels of SREPBs and ATF6 and vice versa. In addition, a significant positive correlation between the levels of *SREBF1* and *SREBF2* was also evident in the tumor samples (P=0.0011, **Figure S1**). In contrast, our analysis revealed no significant correlation between *MBTPS1* and somatostatin (*SST*) or *CREB3* genes (**Figures 1E, F**), and a negative correlation between *MBTPS1* and *BDNF* (**Figure 1G**). Thus far, these data suggest that in the human CRC, MBTPS1 expression levels vary considerably among patients and that this variability is associated with changes in the expression levels of some, but not all of the genes encoding MBTPS1 target proteins.

**Figure 2:**
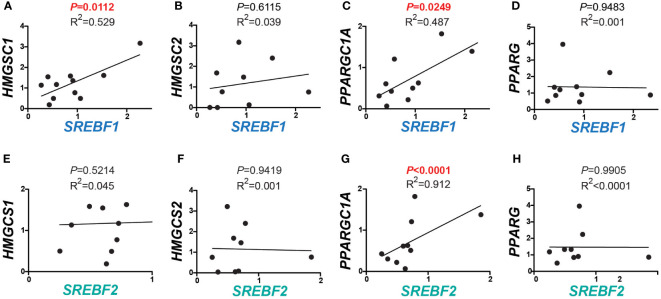
Correlations between expression of SREBFs and their downstream targets in human CRC. **(A–D)** Correlations between *SREBF1* and downstream target genes involved in lipid metabolism. *HMGCS1* and *PPARGC1A* correlated significantly with *SREBF1* (r = 0.7273, *P* = 0.0112, and 0.6978, *P* = 0.0249), while no correlations were detected with *HMGCS2* (r = 0.1970, *P* = 0.6115) and PPARG (r = -0.0236, *P* = 0.9483). **(E–H)** *SREBF2* levels were in correlation with PPARGC1A (r = 0.9552, *P*< 0.0001). No correlations were found between any of *HMGCS1* (r = 0.2127, *P* = 0.4855), *HMGCS2* (r = -0.0285, *P* = 0.9419) and *PPARG* (r = -0.0046, *P* = 0.9905) with *SREBF2*.

Given the positive correlation between the expression of *MBTPS1* and the SREBP encoding genes, we next examined the transcript levels of downstream gene targets of SREBPs within the same tumor samples. As depicted in **Figure 2A**, we found a positive correlation between *SREBF1* and the cytosolic enzyme 3-Hydroxy-3-Methylglutaryl-CoA Synthase 1 (*HMGCS1*), which catalyzes the synthesis of HMG-CoA, a precursor for cholesterol and other products of the mevalonate pathway (3). In contrast, as expected, no correlation was found between the expression levels of *SREBF1* and *HMGCS2*, an HMGSC1 paralog that catalyzes the first step of ketogenesis in the mitochondria to provide lipid-derived energy during starvation (34) (**Figure 2B**). A positive correlation was additionally found between the levels of *SREBF1* and PPARG Coactivator 1 Alpha (*PPARGC1A*) (**Figure 2C**), a transcriptional coactivator of *PPARG* involved in coordination of fatty acid metabolism (35), but not between *SREBF1* and *PPARG* itself (**Figure 2D**). No correlations were found between *SREBF2* and *HMGCS1*, *HMGCS2* (**Figures 2E, F**), or with *PPARG* (**Figure 2H**), but a highly significant correlation was found with *PPARGC1A* (**Figure 2G**, *P*< 0.0001).

### MBTPS1 and its downstream targets regulate the proliferation of colon cancer-derived cells

**Figure 3:**
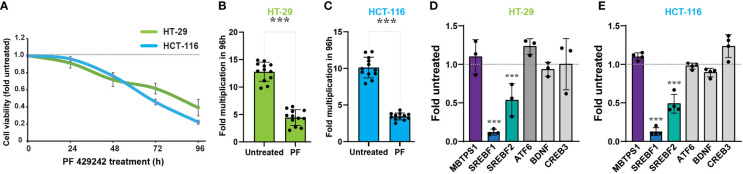
MBTPS1 is involved in proliferation of colon cancer-derived HT-29 and HCT-116 cells. **(A)** Inhibition of MBTPS1 enzymatic activity attenuates cell proliferation. Proliferation of HT-29 cells (green line) and HCT-116 (light blue line) treated with either vehicle or 10 μM PF-429242 for the entire duration of the experiment was measured using the XTT proliferation assay. Shown is an average ± SD of *n*=5 for each condition from four independent experiments. Dotted line represents proliferation of the untreated cells. **(B, C)** Rate of cell multiplication 96h after treatment of HT-29 **(B)** or HCT-116 **(C)** treated with either vehicle or 10 μM PF-429242. *n*=12 Student’s *t*-test ***p<0.0001. **(D)** Relative mRNA levels of *MBTPS1* and its downstream targets 24 hours following treatment with 10 μM PF-429242 in HT-29 cells **(D)** and HCT-116 cells **(E)**. The levels of each gene were compared between PF-429242-treated cells to vehicle. The experiment was conducted in *n*=3 biological repeats with three technical repeats in each experiment. Student’s *t*-test ***p<0.0001.

To determine whether MBTPS1 is directly involved in proliferation of colon cancer cells, we treated two human-derived epithelial adenocarcinoma cells, HT-29 and HCT-116, with a MBTPS1 chemical inhibitor (PF-429242) (36) and measured the effect on cell proliferation. We utilized a concentration of PF-429242 reported as non-toxic to other mammalian cells (37, 38). As shown in **Figure 3A**, attenuation of cell proliferation was evident within approximatly 48 hours of exposure to the MBTPS1 inhibitor and reached 50% at 69.5 and 83 h for HCT-116 and HT-29, respectively. 96 hours after exposure, the rate of cell multiplication was 3-2.5 fold lower in the HT-29 and HCT-116 cells treated with PF-429242 compared to their controls, respectively (**Figures 3B, C**).

We next measured the transcript levels of *MBTPS1* and its downstream gene targets in HT-29 and HCT-116 cells following 24 hours of treatment with PF-429242, reasoning that the change in gene expression preceeds that of proliferation. As depicted in **Figures 3D, E**, inhibition of MBTPS1 did not cause a significant change in its mRNA levels in neither cell line, nor was there a change in the mRNA levels of *CREB3*, *BDNF* and *ATF6*. The most significant effect of PF-429242 treatment was observed on the mRNA levels of *SREBF1* (12% compared to the vehicle-treated cells in both lines) and in *SREBF2* (54- and 45% compared to the vehicle-treated HT-29 and HCT-116 cells, respectively). Of note is the finding that SST levels in both cell lines were below detection, suggesting that this gene does not play a significant role in the effect of MBTPS1 inhibition on proliferation of either cell line.

**Figure 4:**
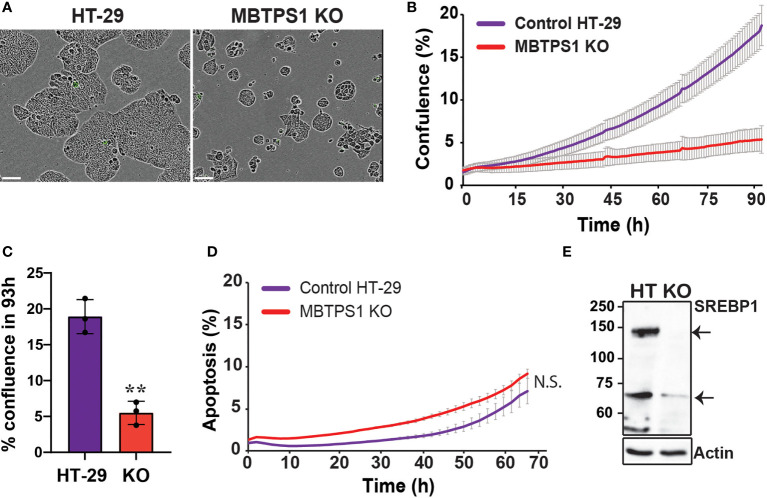
CRISPR/Cas9-mediated knockout of the MBTPS1 gene attenuates cell proliferation of colon cancer-derived HT-29 cells. **(A)** Representative images of original HT-29 and MBTPS1 KO cells. Scale bar represents 300 mm. **(B)** Knockout of MBTPS1 in HT-29 cells leads to attenuated proliferation. Shown is a representative of six independent experiments tracking the growth of original HT-29 and MBTPS1 KO cells, average ± SD of *n*=3 for each condition. **(C)** % confluence of original HT-29 and MBTPS1 KO cells 96 h after seeding (*n*=3, t-test, **P<0.001). **(D)** Staining of original HT-29 and MBTPS1 KO cells with the active caspase 3/7 reagent did not show significant differences in the percentage of apoptosis between the two cell lines. Shown is an average ± SD of *n*=2 for each condition. **(E)** A representative immunoblot of control HT-29 and MBTPS1 KO cells stained for SREBP1 showing a significant reduction in the expression of SREBP1 precursor (125 kD, top arrow) and mature SREBP1 (68 kD, bottom arrow).

The dramatic effect of the MBTPS1 inhibitor, PF-429242, on proliferation of CRC cell lines suggested that elimination of the *MBTPS1* gene may slow down cell proliferation, or even halt it completely. However, we could not rule out that PF-429242 has also MBTPS1-independent effects on cell division. To this end, we attempted a CRISPR/Cas9-mediated knockout (KO) of the three alleles of the *MBTPS1* gene in HT-29 cells (**Figure S2**). Following this intervention and despite analysis of hundreds of clones, we succeeded in identifying only one clone in which the three allelic copies of *MBTPS1* were disrupted (**Figure S2C**). Accordingly, *MBTPS1* mRNA levels in this clone were reduced to less than 10% of the original cell line (**Figure S2D**). Consistent with the effect of the chemical inhibitor, KO of *MBTPS1* expression resulted in marked retardation of approximately 5-fold in cell proliferation rate (**Figures 4A–C**). The MBTPS1 KO cells showed similar levels of annexin V-positive cells as control cells (6-7%) (**Figure 4D**), suggesting that the retarded proliferation in cells lacking *MBTPS1* expression is not due to cell death. Furthermore, SREBP1 protein levels were significantly reduced by MBTPS1 KO (**Figure 4E**). Collectivity, genetic and pharmacological inhibition in colon cancer-derived HT-29 and HCT-116 cells indicate that MBTPS1 plays an essential role in proliferation of cells of this cancer type.

Pharmacological inhibition and genetic manipulation independently and consistently show that MBTPS1 plays a critical role in CRC-derived cell proliferation. Therefore, we postulated that the single *MBTPS1*-KO clone isolated following our CRISPR/Cas9 manipulation could serve to uncover both the cellular pathways affected by the absence of *MBTPS1* as well as pathways that may be upregulated to enable survival of this single KO clone. To that end, we first performed RNA-seq analysis on the control and MBTPS1 KO HT-29, which identified 3,391 genes that were differentially expressed (DE) between the two lines (FDR threshold *P*<0.05). Among these genes, 1,671 (49%) were upregulated and 1,720 (51%) were downregulated in the *MBTPS1*-KO cells compared to the unmanipulated HT-29 cells (Benjamini-Hochberg adjusted *P* value<0.05) (**Table S1**) SREBF1 and SREBF2 were among the downregulated genes, confirming the observations in (**Figures 3**, **4**) that inhibition of MBTPS1 leads to a significant reduction in SREBFs transcript and protein levels. 

**Figure 5:**
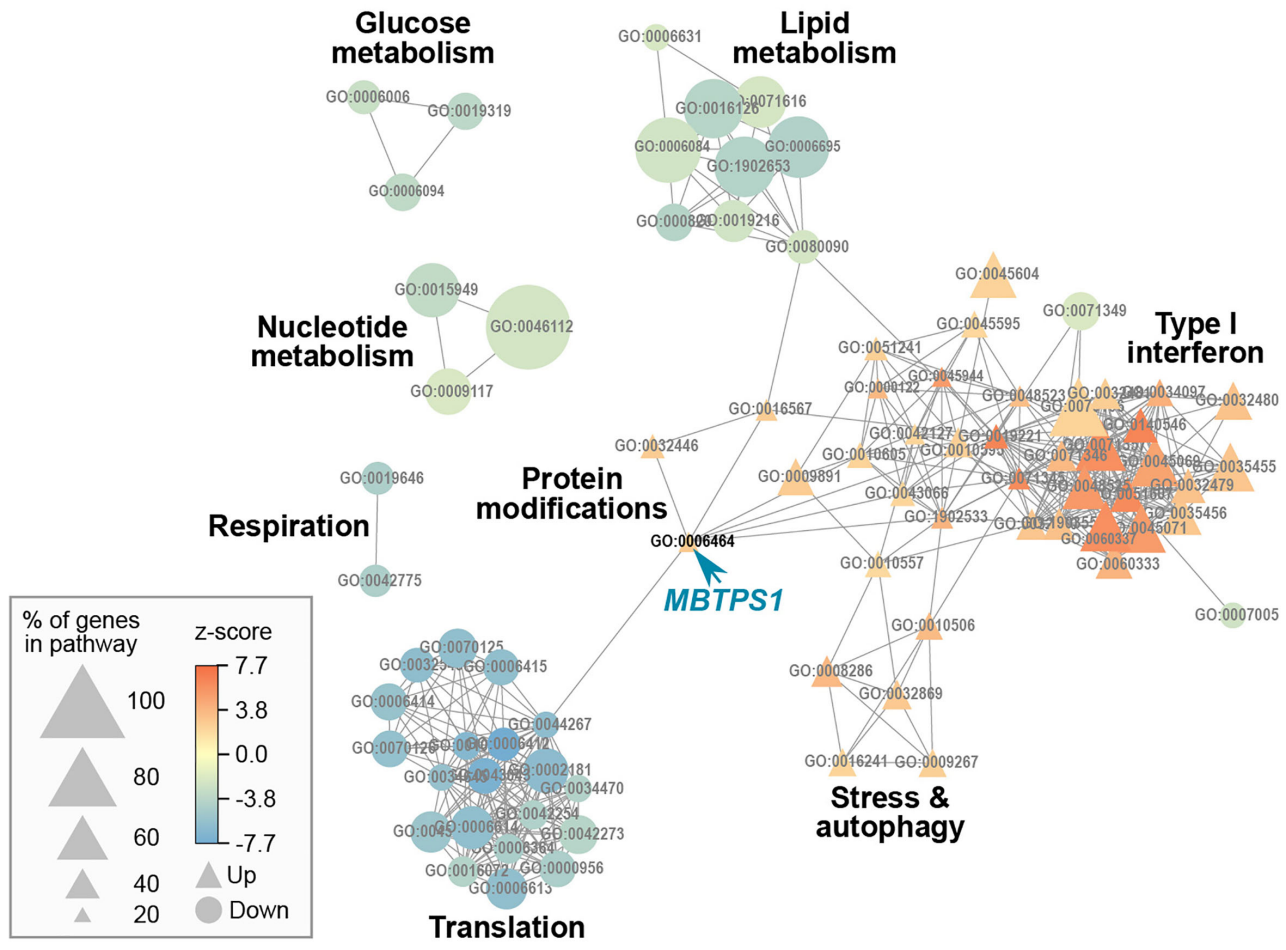
Gene ontology network of MBTPS1 KO. Overlap between pathways that are significantly enriched in the DE gene set of MBTPS1 KO compared to original HT-29 cells. GO terms are represented as nodes. The size of the node indicates the percentage of genes in the pathway that are DE. The node color represents the z-score, which calculates the trend of DE genes toward increased (Up 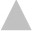) or decreased (Down 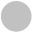) expression.

We then applied a z-score cutoff of 2 (absolute value) and performed a gene ontology (GO) analysis, which resulted in a list of 87 pathways. Since many of the genes are common to more than one pathway, we calculated a pathway network describing this overlap, which resulted in eight network modules (**Figure 5**). In such a depiction, a positive z score reflects GO terms in which most of the genes were elevated, and vice versa for a negative z score. *MBTPS1*, whose level is significantly reduced in the KO cells (**Figure S2**), appears in the “cellular protein modification process” term (GO:0006464), a large term that contains 1,025 genes, 223 of which are affected in the KO cells. Since this term contains slightly more upregulated than down regulated terms, it is depicted as having a positive z score. This module interconnects the largest identified modules (lipid metabolism, translation and type-1 interferon), and consistent with the patient data, MBTPS1 KO also has a marked effect on modules that include SPEBFs and ATF6 (GO:0045944).

### MBTPS1 knockout upregulates the type-1 interferon pathway

In our analysis, the most dramatic effect of MBTPS1 KO appears in the form of upregulation of the type-1 interferon (type-1 IFN) pathway (**Figure 5**). For example, in the cellular response to type-1 IFN (GO:0071357), 34 of the 65 genes (52%) in the pathway were differentially expressed, all of which were upregulated. Similarly, in the regulation of type-1 IFN production (GO:0032479), 32 of the 89 genes (36%) in the pathway were affected, 25 of which were upregulated (**Figure 5** and **Table S1**).

**Figure 6:**
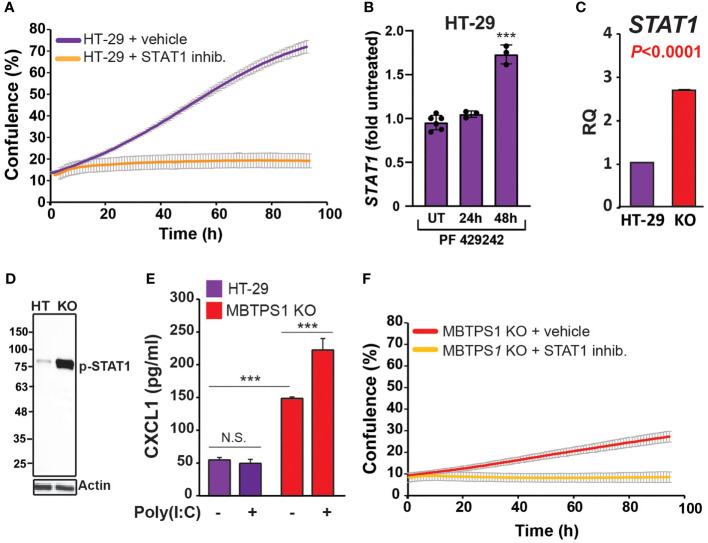
MBTPS1 knockout upregulates the type-1 interferon pathway. **(A)** STAT1 activity is critical for proliferation of HT-29 cells. Proliferation of HT-29 cells treated with either vehicle or 25 mM STAT1 inhibitor fludarabine was tracked over 96 h using time-laps microscopy. Shown is an average ± SD *n*=3 for each condition. **(B)** Relative STAT1 mRNA levels 24 and 48 hours following treatment with 10 mM PF-429242 in HT-29 cells. *n*=3-6 One –Way ANOVA ***p<0.0001. **(C)** Relative mRNA levels of STAT1 in the original and MBTPS1 knockout HT-29 cells (*n*=3, t-test, P<0.0001). The levels of STAT1 in the knockout are markedly elevated, in accordance with the transcriptome data. **(D)** Immunoblot of control HT-29 and MBTPS1 KO cells show elevated levels of phosphorylated STAT1 (p-STAT1). Representative blots of n=3. **(E)** MBTPS1 KO cells show a functional response to poly(I:C) stimulation. Shown is CXCL1 production in response to stimulation of HT-29 or MBTPS1 KO cells with 50 mg/ml poly(I:C) overnight (*n*=4 repeats). **(F)** Proliferation of MBTPS1 KO cells treated with either vehicle or 25 mM STAT1 inhibitor fludarabine. Shown is an average ± SD n=3 for each condition. N.S., not significant.

Since several studies have indicated the existence of type-1 IFN pathway in HT-29 cells (39, 40), we first tested whether inhibition of STAT1, one of the key downstream components of this pathway upregulated in the MBTPS1 KO transcriptome, affects the proliferation of the original HT-29 cells. As depicted in **Figure 6A**, inhibition of STAT1 caused a significant arrest in proliferation, indicating that the type-1 IFN pathway is critical for proliferation of these cells. Next, we tested whether inhibition of MBTPS1 activates the type-1 IFN pathway, by measuring the transcript levels of STAT1 in the presence of the MBTPS1 inhibitor. As shown in Figure 6B, MBTPS1 inhibition did not affect the levels of STAT1 mRNA during the first 24 hours of treatment. Notably, at that time point the levels of SREBFs were already markedly downregulated by PF-429242 (**Figure 3**), but there was still no apparent effect on proliferation (**Figure 3A**). However, by 48 hours of treatment, proliferation was already affected (**Figure 3A**), many cell died and the remaining ones showed marked upregulation of STAT1 (**Figure 6**). This confirmed that MBTPS1 inhibition leads to activation of the IFN pathway, which occurs after its effect on SREBPs.

Given the above findings, we tested the hypothesis that the sole MBTPS1 KO clone survived due to permanent upregulation of type-1 IFN pathway. In support, we found that STAT1 mRNA levels increased by almost 3-fold in the MBTPS1 KO cells compared to the control HT-29 cells (**Figure 6C**). Accordingly, the protein levels of phospho-STAT1 were markedly elevated in the knockout cells (**Figure 6D**). To confirm that the increase in STAT-1 mRNA and protein levels reflects an increase in a functional interferon system, we challenged control and MBTPS1 KO HT-29 cells with polyinosinic:polycytidylic acid (Poly(I:C)), an immune-stimulant that mimics viral infection (41), and measured the levels of CXCL1, one of the chemokines generated in response to this type of challenge. Measurement of CXCL1 following exposure to Poly(I:C) revealed that the original HT-29 cells lack the ability to respond to Poly(I:C) stimulation. In contrast, MBTPS1 KO cells had a significantly higher basal level of CXCL1 compared to the original HT-29 line and responded by further elevation of its levels in response to the Poly(I:C) stimulation (**Figure 6E**). Finally, the application of the STAT1 inhibitor arrested the proliferation of the MBTPS1 KO cells entirely (**Figure 6F**), providing additional support to the hypothesis that the MBTPS1 KO clone indeed survived due to upregulation of the type-1 IFN pathway. 

### *MBTPS1 affects cell proliferation primarily through* the SREBP pathway

**Figure 7:**
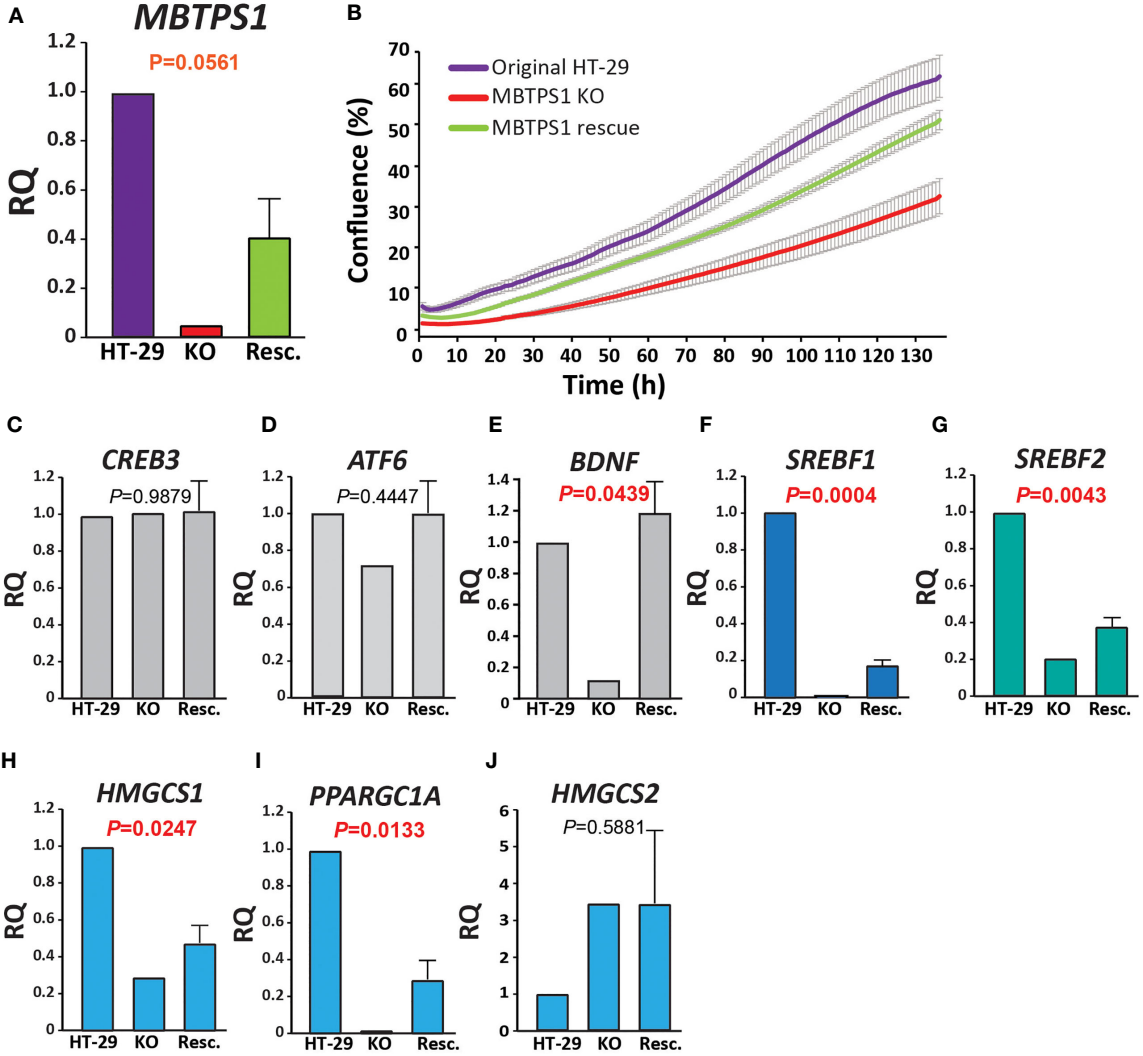
MBTPS1-mediated effect on proliferation is dependent mainly upon the SREBP pathway. **(A)** Relative mRNA levels of *MBTPS1* in original (HT-29), MBTPS1 KO and re-expression of MBTPS1 (Rescue) HT-29 cells. *P*-values indicate a significant difference between KO and Resc. HT-29 cells Reintroduction of the *MBTPS1* gene into the KO cells resulted in partial recovery of its mRNA levels (*n*=4). **(B)** Proliferation of HT-29, MBTPS1 KO and MBTPS1 rescued cells was tracked using time-lapse microscopy. Shown is an average ± SD of *n*=3 for each condition from four independent experiments. In accordance with the partial recovery in MBTPS1 expression, the rate of proliferation of the cells with ectopic MBTPS1 expression was intermediate between the original and the KO cells. **(C, D)** No significant effect of MBTPS1 rescue on the relative mRNA expression of MBTPS1 targets *CREB3* and *ATF6* (*P*=0.09879 and 0.4447, respectively). **(E)** Significant reduction in the expression of *BDNF* in MBTPS1 KO cells and its complete recovery in the cells with ectopic MBTPS1 expression (*P*=0.0439). **(F, G)** Significant effect of MBTPS1 expression on its downstream targets *SREBF1* (*P*=0.0004) and *SREBF2* (*P*=0.0043). The levels of both targets drop significantly and show a small but significant recovery. **(H, I)** Significant reduction and partial recovery of the SREBP downstream targets *HMGCS1* (*P*=0.0247) and *PPARGC1A* (*P*=0.0133). **(J)** The absence of MBTPS1 causes a marked elevation in *HMGSC2* that is not restored to normal levels after partial recovery of MBTPS1 (*P*=0.5881), suggesting that part of the transcriptional changes that occurred in the MBTPS1 KO cells are irreversible. *n*=2-4 repeats, One-Way ANOVA, *P*<0.05 significant.

In addition to upregulation of the type-1 IFN pathway, MBTPS1 KO was accompanied by a marked reduction in the expression of genes in five modules: glucose metabolism, lipid metabolism, nucleotide metabolism, respiration and translation (**Figure 5**). Of the modules that are known to be directly linked to MBTPS1, the cholesterol biosynthetic process (GO:0006695) is highlighted as the functional group with the highest changes in expression: 63% of the genes in this module are altered in the MBTPS1 KO cells, 95% of which are downregulated. In order to verify that these phenotypes are directly related to MBTPS1 KO, we reintroduced the wildtype MBTPS1 gene into the MBTPS1 KO cells and examined whether the abnormal phenotypes in these cells are rescued. Ectopic expression of *MBTPS1* in MBTPS1 KO cells only partially restored *MBTPS1* transcript levels, albeit, not to that of the original HT-29 cells (**Figure 7A**). In accordance with the partial rescue in MBTPS1 expression, the proliferation rate of the rescued cells was also partially restored to an intermediate level between the original and KO HT-29 cells (**Figure 7B**). Subsequent measurements of MBTPS1 targets revealed that *CREB3* and *ATF6* mRNA levels were not significantly affected by re-expression of *MBTPS1* (**Figures 7C, D**). *BDNF* levels were eliminated almost completely in the MBTPS1 KO cells but since they recovered completely upon reintroduction of *MBTPS1* (**Figure 7E**), they probably do not play a significant role in MBTPS1-mediated regulation of HT-29 cell divisions, or their role is not reflected in changes in mRNA levels.

In agreement with the transcriptome analysis, RT-qPCR analysis revealed that the levels of both SREBFs were significantly reduced by MBTPS1 KO. In particular, *SREBF1* expression was completely lost in MBTPS1 KO cells. Despite the partial recovery in *MBTPS1* levels, the recovery of *SREBF1* expression was minor but significant (**Figure 7F**). *SREBF2* levels were also markedly affected by MBTPS*1* knockout and recovered partially, albeit to higher levels than *SREBF1* (**Figure 7G**). Partial recovery was also observed in mRNA levels of the downstream affected genes *HMGCS1* and *PPARGC1A* (**Figures 7H, I**). Remarkably, knockout of *MBTPS1* caused a marked elevation in *HMGCS2* levels (**Figure 7J**), which was not reduced back to normal levels following the reintroduction of *MBTPS1*, suggesting that despite the partial re-expression of *MBTPS1*, the cells continued to display an energy-deprived phenotype.

## Discussion

Here, we investigated the direct involvement of MBTPS1 in colon cancer by integrating data from human CRC tumors and *in vitro* models of CRC-derived cell lines. The dataset obtained from patients with colorectal cancer provided valuable information regarding significant correlations between MBTPS1 expression and some, but not all, of its downstream targets. However, it did not offer mechanistic information regarding the role of MBTPS1 in proliferation. Conversely, manipulating MBTPS1 activity in the CRC-derived cell lines provided insights into its role in CRC proliferation. A previous study has indicated that other PCs, furin and PC5A enhance proliferation of HT-29 cells by cleavage of the IGF-1 receptor (42). By combining the information derived from the two datasets herein, we conclude that another PC, MBTPS1 plays a significant role in regulating proliferation of colorectal cancer cells.

MBTPS1 has several known substrates. In order to identify which of them may be involved in CRC proliferation we used a combined strategy of patient and cell-derived data. Using this approach we found that while CREB3, SST and BDNF may be involved in CRC, they are not the downstream mediators of MBTPS1 on proliferation. Surprisingly, ATF6, a well-known target of MBTPS1 whose expression levels were found to be in strong correlation with those of MBTPS1 in the patient samples, does not play a significant role in the MBTPS1-mediated regulation of CRC-derived cell proliferation (**Figures 3**, **7**). However, studies show that ATF6 may also be activated by specific lipids (16), which may explain the correlation between ATF6 and MBTPS1 levels in CRC tumors.

The SREBP1/2 pathway was the only one that showed similar trends in the tumors and the MBTPS1 KO cells, results which are in agreement with earlier findings which showed that downregulation of SREBPs inhibits tumor growth in colon cancer (20). In the tumors, there was a strong positive correlation between the expression of *MBTPS1* and both *SREBPs* (**Figures 1A, B**), and between *SREBP1* and its downstream targets (**Figures 2A, C**). Interestingly, despite the strong correlation between expression levels of *SREBP1* and *SREBP2* (**Figure S1**), no correlation was detected between expression of *SREBP2* and *HMGCS1* in tumors (**Figure 2E**), suggesting that the effect on *HMGCS1* is mediated *via* SREBP1, but not SREBP2. Furthermore, no correlations were evident between either SREBPs and HMGCS2, that regulates ketogenesis (43), or with PPARG, that is activated by PPARGC1A (44). The most significant change in the MBTPS1 KO cells also occurred in the SREBP pathway. The expression of both SREBPs, and especially SREBP1, was significantly reduced in the absence of MBTPS1 (**Figures 7F, G**), and the transcript levels of downstream targets of SREBPs, HMGCS1 and PPARGC1A, were also downregulated to a great extent (**Figures 7G–I**). Interestingly, MBTPS1 KO upregulated HMGCS2 mRNA levels by more than 3-fold, suggesting that the MBTPS1 KO cells display a phenotype of fasting cells. In contrast to partial recovery of other downstream targets following ectopic expression of MBTPS1, HMGCS2 expression remained elevated (**Figure 7J**), suggesting that part of the changes in the MBTPS1 KO cells are secondary to MBTPS1 elimination and are irreversible.

The results presented in this study demonstrate that impairment of MBTPS1 activity or KO of its gene is detrimental to CRC cells. This is best illustrated by the fact that despite our vast attempts, we obtained only one clone that proliferated following the KO of the three *MBTPS1* alleles in HT-29 CRC cells. Based on findings from the gene expression analysis of this KO clone as well as additional experiments, we showed that the survival of this clone is most likely due to upregulation of the type-1 IFN pathway. Importantly, HT-29 cells depend on this pathway for their proliferation (**Figures 6A, B**), but in the MBTPS1 KO cells this pathway was upregulated even further. An interplay between the immune system and lipid synthesis was reported previously, although in contrast to our system, it involved signaling between more than a single cell type. A prominent study identified a crucial role for SREBPs in regulating the intra-tumor response of Regulatory T cells (Treg), which drive immunosuppression in the tumor microenvironment (45). In this latter study, inhibition of SREBP-dependent lipid synthesis caused reprogramming of Treg cells such that it enabled an effective anti-tumor immune response by other cells within the tumor environment. In our study, the reduction or absence of MBTPS1 in CRC cells downregulated SREBPs and upregulated the intracellular type-1 IFN system. Furthermore, the levels of STAT1, which increased significantly in the KO cells (**Figure 6A**), remained high even after re-introduction of MBTPS1 into the cells (2.38 + 0.7 fold). Together these findings suggest that upregulation of type-1 interferon response enabled the proliferation of CRC cells despite the loss of MBTPS1. Further studies are required to understand how the mechanistic relationship between these two pathways.

MBTPS1 KO cells were grown in a nutrient-rich environment that contains high glucose levels and is supplemented with fetal bovine serum that includes lipids, carbohydrates, protein and cholesterol (46). Nonetheless, these cells presented with a phenotype of hunger and low energy, reflected by high HMGSC2 levels that provide lipid-derived energy during carbohydrate deprivation (**Figure 7J**). Since cholesterol synthesis and uptake are regulated at the transcriptional level by SREBPs, it is plausible that the levels of key enzymes such as HMG CoA reductase (the rate-limiting enzyme for cholesterol biosynthesis) and LDLR, which mediates endocytosis of cholesterol-rich LDLs (47), are affected by MBTPS1 KO, thus causing imbalance in lipid metabolism that affects additional central metabolic pathways including glycolysis and the respiratory electron transport chain.

In conclusion, using a combined chemical and CRISPR/Cas9 –gene knockout approach we show that MBTPS1 plays a pivotal role in proliferation of colon cancer cells. Furthermore, by comparing data from human CRC samples to that of CRC-derived cell lines we were able to rule out the involvement of certain downstream MBTPS1 targets in regulating proliferation, and to identify the SREBP pathway as most likely responsible for this effect. Nonetheless, the data herein only studies the effect of MBTPS1 KO on cell proliferation. Thus, we cannot completely rule out the possibility that the other MBTPS1 targets affect additional cellular functions that may have an indirect effect on cell survival. Whether MBTPS1 inhibition may serve as an additional therapeutic target in CRC remains to be determined.

## Data availability statement

The datasets presented in this study can be found in online repositories. The names of the repository/repositories and accession number(s) can be found in the article/**Supplementary Material**.

## Ethics statement

The studies involving human participants were reviewed and approved by Institutional Review Board of Ha’Emek Medical Center (protocol code 0049–19-EMC 6 January 2022). The patients/participants provided their written informed consent to participate in this study.

## Author contributions

Conceptualization, SS and LB-H; Data curation, ML; Formal analysis, LH-B, ES, STo, ML, OAH, STa, SS, and LB-H; Funding acquisition, OAH and LB-H; Investigation, LH-B, ES, STo, LS, OAH, STa, and SS; Methodology, SS and LB-H; Project administration, STa and LB-H; Resources, LB-H; Supervision, SS and LB-H; Validation, ML; Visualization, STa and LB-H; Writing – original draft, SS and LB-H; Writing – review & editing, LH-B, STo, SS, and LB-H. All authors contributed to the article and approved the submitted version.

## Funding

This work was supported by the Israel Science foundation [#1445/15 & 2240/19] and by the Israel Cancer Association [#20210063] to LB-H.

## Acknowledgments

We thank Ms. Liran Giladi for technical assistance, Dr. Amiram Ariel for materials and discussion regarding the type-1 interferon pathway, and Drs. Martin Mikl, Amir Sapir and Hila Toledano for critical manuscript comments.

## Conflict of interest

The authors declare that the research was conducted in the absence of any commercial or financial relationships that could be construed as a potential conflict of interest.

## Publisher’s note

All claims expressed in this article are solely those of the authors and do not necessarily represent those of their affiliated organizations, or those of the publisher, the editors and the reviewers. Any product that may be evaluated in this article, or claim that may be made by its manufacturer, is not guaranteed or endorsed by the publisher.
