# Supplementary material for: MBTPS1 regulates proliferation of colorectal cancer primarily through its action on sterol regulatory element-binding proteins
Source: Front Oncol. 2022 Oct 10;12:1004014. doi: 10.3389/fonc.2022.1004014 (PMC9592115; doi:10.3389/fonc.2022.1004014)
Supplement: Supplementary file 2 [file DataSheet_2.docx]

**
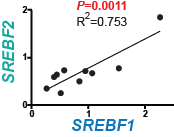
**

**Figure S1**. The correlation between expressions of SREBPs in human CRC. *SREBF1* mRNA levels correlated significantly with those of *SREBF2* (r = 0.6171, *P* = 0.0011).


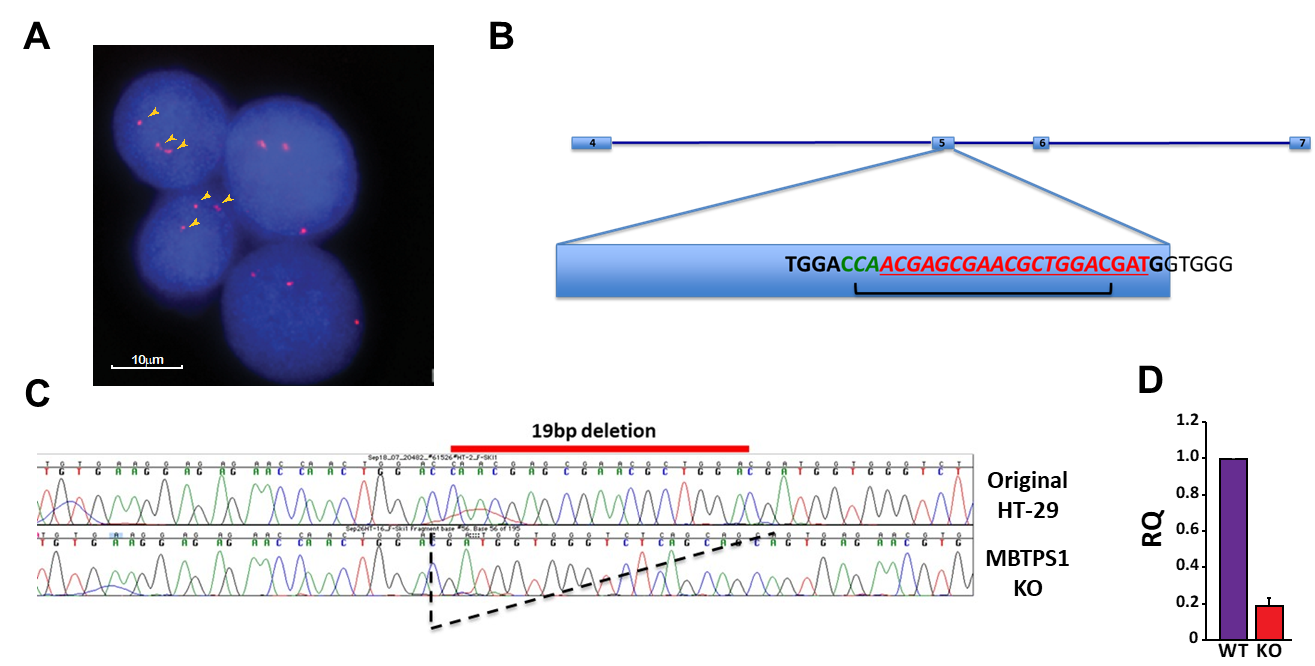


**Figure S2.** Disruption of the *MBTPS1* gene in HT-29 cells by CRISPR/Cas9 editing. (**A**) HT-29 cells contain three alleles of the *MBTPS1* *gene*. To determine the number of *MBTPS1* gene copies in HT-29 cells, FISH was carried out with a BAC from the SKI1 region on HT-29 nuclei. Arrows point to FISH signals in nuclei. (**B**) A map describing the exon/intron structure of the *MBTPS1* gene between exons 4-7. The guide RNA target sequence is positioned in exon 5 (red underlined sequence). The NGG PAM sequence is depicted in green. The guide binds to the antisense DNA strand. The exonic sequence surrounding the guide binding site is in bold. The 5’ end of intron 5 appears in non-bold letters. (**C**) The *MBTPS1* KO clone contains a 19-nucleotide deletion in the open reading frame of one of the three alleles. Exon 5 was amplified from HT-29 and from the KO clone with primers positioned in introns 4 and 5. The chromatograms show the region of the 19-nucleotide deletion. The deleted region is marked also in (**B**). An Identical deletion was identified by sequencing cDNA products amplified with primers positioned to exons 4 and 7. The PCR result indicates that two out of the three alleles of the gene were not amplified due to CRISPR/Cas9 induced deletions that included one or both of the PCR primer binding sites (**D**) Quantitative RT-PCR using Sybr Green (See Materials and Methods) indicates that *MBTPS1* expression in the KO clone is reduced by 80- 90%.
